# Super-resolution high-speed optical microscopy for fully automated readout of metallic nanoparticles and nanostructures

**DOI:** 10.1038/s41598-020-75883-z

**Published:** 2020-11-02

**Authors:** Andrey Alexandrov, Takashi Asada, Giovanni De Lellis, Antonia Di Crescenzo, Valerio Gentile, Tatsuhiro Naka, Valeri Tioukov, Atsuhiro Umemoto

**Affiliations:** 1 I.N.F.N. Sezione di Napoli, 80126 Napoli, Italy; 2 Università degli Studi di Napoli Federico II, 80126 Napoli, Italy; 3 National University of Science and Technology MISIS, 119049 Moscow, Russia; 4 Lebedev Physical Institute of the Russian Academy of Sciences, 119991 Moscow, Russia; 5 I.N.F.N. LNGS-Laboratori Nazionali del Gran Sasso, Assergi, 67100 L’Aquila, Italy; 6 CERN, Geneva, Switzerland; 7 Kobayashi-Maskawa Institute for the Origin of Particles and the Universe, Center for Experimental Studies, Nagoya University, Furou-cho, Chigusa-ku, Nagoya, 464-8602 Japan; 8 Department of Physics, Toho University, Funabashi, Chiba 274-8510 Japan; 9 Graduate School of Science, Nagoya University, Furo-cho, Chikusa-ku, Nagoya, 464-8602 Japan

**Keywords:** Polarization microscopy, Super-resolution microscopy, Nanometrology, Characterization and analytical techniques, Imaging techniques, Microscopy, Characterization and analytical techniques, Imaging techniques, Microscopy, Liquid crystals, Dark energy and dark matter

## Abstract

We have designed a fully automated optical microscope running at high-speed and achieving a very high spatial resolution. In order to overcome the resolution limit of optical microscopes, it exploits the localized surface plasmon resonance phenomenon. The customized setup using a polarization analyzer, based on liquid crystals, produces no vibrations and it is capable of probing isolated nanoparticles. We tested its performance with an automated readout using a fine-grained nuclear emulsion sample exposed to 60 keV carbon ion beam and, for the first time, successfully reconstructed the directional information from ultra-short tracks produced by such low-energetic ions using a solid-state tracking detector.

## Introduction

The demand for advanced imaging of nanometer-scale objects at a spatial resolution below the diffraction limit has led to the appearance of various super-resolution techniques. Many of them, such as stimulated emission depletion (STED) microscopy^1^, spontaneous emission and photoactivated localization microscopy (PALM)^2^, stochastic optical reconstruction microscopy (STORM)^3^, take advantage of optical properties of fluorescent emitters to selectively switch on or off close molecules or nanoparticles (NPs). Then, it becomes possible to use barycenters of isolated images, thus, providing a better resolution. Recently reported orientation-dependent localization microscopy (ODLM)^4^ and super-resolution plasmonic imaging microscopy (SRPIM)^5^ both exploit the localized surface plasmon resonance (LSPR) phenomenon^6^ for imaging of non-fluorescent metallic NPs in dielectric media.

Plasmon resonance is the collective oscillation of free electrons induced by an external electric field^6^. Free electrons in metallic NPs have a natural frequency due to the binding force responding to displacement generated by electrostatic attraction in an external electric field. Silver NPs with dimensions from few tens to few hundreds of nanometers undergo a resonance condition at visible wavelengths. In addition, if assuming a non-spherical structure, like an ellipsoid body, the dipole moment depends on the external electric field direction, which can be detected by analysing the angle of linear polarization of the reflected light^6^. Thus, plasmon resonance provides information at smaller spatial scales than the standard optical imaging.

The nuclear emulsion^7,8^ is composed of tiny silver bromide (AgBr) crystals immersed in a gelatin binder. The crystals act as sensors that are activated by the ionization loss of a passing-through charged particle. The activated state of the crystals is preserved until the emulsion film is chemically developed. Thus, a particle track is recorded, first as a sequence of activated crystals, which later, after the development, becomes a sequence of silver NPs, called grains^8^. These grains have the form of randomly oriented filaments with typical dimensions equal to several tens of nanometers, depending on the emulsion type^8^.

The new type of a fine-grained emulsion, called the Nano-Imaging Tracker (NIT)^9^, was specifically designed to be used as a detector in the Nuclear Emulsion WIMP Search with directional measurement (NEWSdm) experiment^10^. This experiment develops the next-generation strategy for directional detection^11,12^ of the so-called Weakly Interacting Massive Particle (WIMP)^13–15^ with a complementary approach that provides unambiguous signature of the galactic origin of dark matter.

With using the SRPIM technique it was demonstrated the feasibility of reconstructing tracks with lengths shorter than 315 nm^5^, the optical resolution of the microscope in green light. These tracks were produced by low-energy (100 keV) carbon ions in an NIT emulsion film. In order to reach and, eventually, overcome the so-called neutrino floor^16^ the NEWSdm detector must have a high sensitivity and a sufficiently large mass: from 10 to 100 tons, depending on the sensitivity^16^. The major fraction of events are expected to have track lengths shorter than 200 nm. Therefore, to have a higher sensitivity and also the sensitivity for low-mass WIMPs $(<10\,\hbox {GeV}/\hbox {c}^2)$, the detection threshold must be as low as possible^16^. Such a low threshold requires a super-resolution that, combined with the necessity to analyse several tons of the NIT emulsion, makes the readout an extremely challenging task, impossible without a creation of a high-speed fully automated super-resolution microscopes, like the one we report in this paper.

In the field of nuclear emulsion analysis sub-micron-length tracks, produced by low-energetic charged particles and composed of silver nano-grains, with typical distances between them being shorter than the optical resolution $(\lesssim {200}\, \hbox {nm})$ are referred to as *nanotracks* to distinguish them from other track types that can appear in nuclear emulsion, for example, microtracks, black tracks, etc. Nevertheless, in view of the fact that all the tracks we consider in this paper are nanotracks and to avoid reader’s confusion, we do not call them nanotracks but tracks.

**Figure 1:**
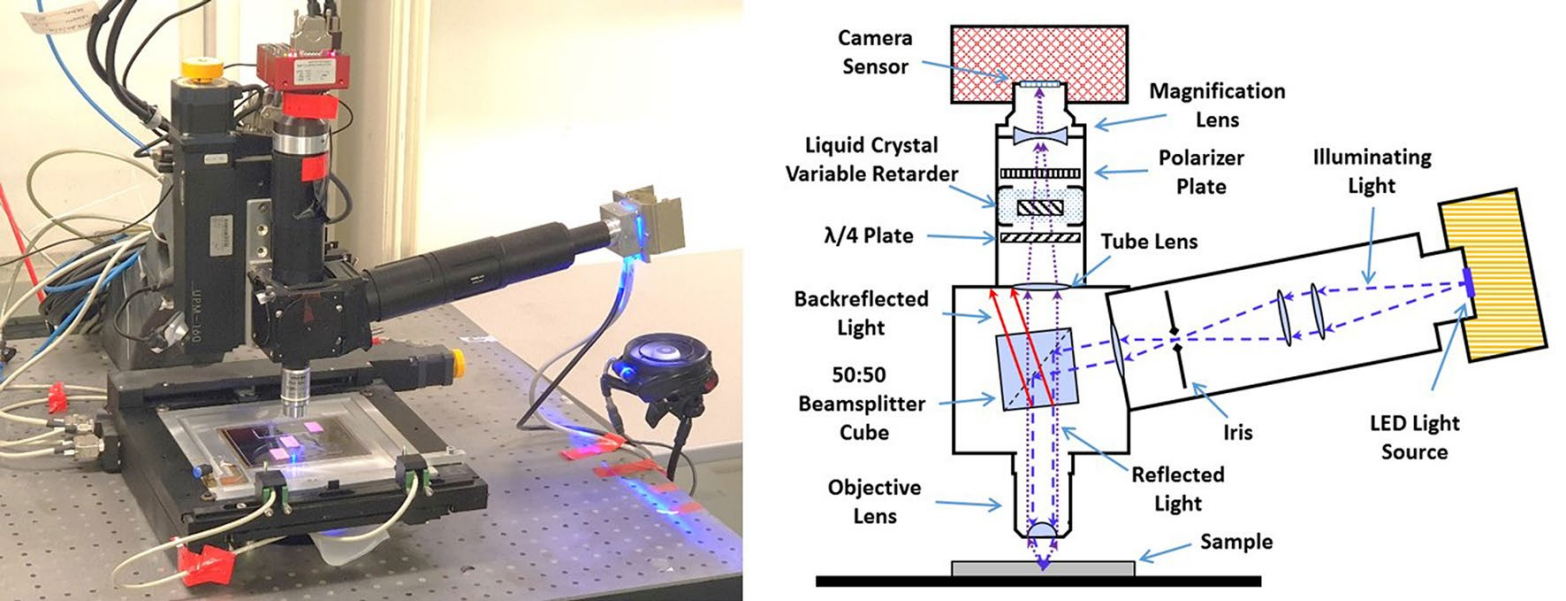
A picture of the developed microscope prototype (left) and the technical scheme of its setup (right).

## Results

### Microscope setup

The microscope, designed and used to carry out the measurements, is shown in the left panel of Fig. 1. It uses a high magnification objective lens $(100\times )$ with high numerical aperture $(NA=1.45)$. Combined with an auxiliary magnification lens, the effective microscope magnification becomes $260\times$. In order to improve the optical contrast the illumination system works in the reflected light mode and houses a bright LED illumination source with $\lambda = 460 \pm 25\,\hbox {nm}$.

We have chosen to use a beamsplitter cube since, unlike a semi-transparent mirror, it does not noticeably distort the polarization of neither the passing-though nor the reflected light. In order to prevent the backreflected light, produced by the faces of the beamsplitter cube, from entering the tube lens and reaching the camera, we have inclined the beamsplitter cube by about $5^{\circ }$, as shown in the scheme the right panel of Fig. 1.

The LED has a uniform intensity distribution across the whole emitting surface of $3 \times 4\,\hbox {mm}^2$, making the use of the so-called critical illumination possible. Tests have shown that, in our case, the critical illumination provides 3–4 times better contrast with respect to the more widespread Kohler illumination.

The microscope is equipped with a fast 4M monochromatic camera with $7 \upmu \,\hbox {m}$ sensor pixel size. The digital resolution is 27 nm/pixel making the field of view as large as $65\,\times \,48 \upmu \,\hbox {m}^2$. The optical resolution, measured by fitting the point spread function of spherical silver NPs, is equal to $230 \pm 10\, \hbox {nm}$.

### Liquid crystal polarization analyzer

All readouts reported so far are implemented by means of mechanical rotation of either an optical element, like the polarization filter^5,17^, or the sample itself^4^, inevitably producing noticeable vibrations. Mechanical rotation followed by some idle time to dump vibrations would not only slow down the readout but, potentially, can also lead to localization errors due to the sample drift between consecutive polarization angle measurements. Both vibrations and sample drift, if not corrected, interfere with measurements spoiling the spatial accuracy and, hence, limiting the achievable resolution.

We have designed a polarization analyzer containing no moving parts. Its core is a liquid crystal variable retarder (LCVR), an electrooptical device exploiting anisotropic optical properties of nematic liquid crystals (LC). The retardancy introduced into the light beam passing through a LC cell depends on the orientation of LC molecules with respect to the light beam direction: the retardancy is maximal when the molecules are aligned with the light beam. The orientation of molecules, which are in fact electric dipoles, can be controlled by applying an external electric field to the transparent faces of the LC cell. The rotation of molecules is quite fast and is of the order of 10–20 ms, depending on the LC cell thickness. Therefore, the performance of the designed LC polarization analyzer (LCPA) is equivalent to an ordinary polarization filter vibrationlessly rotating at about 200–400 rpm.

**Figure 2:**
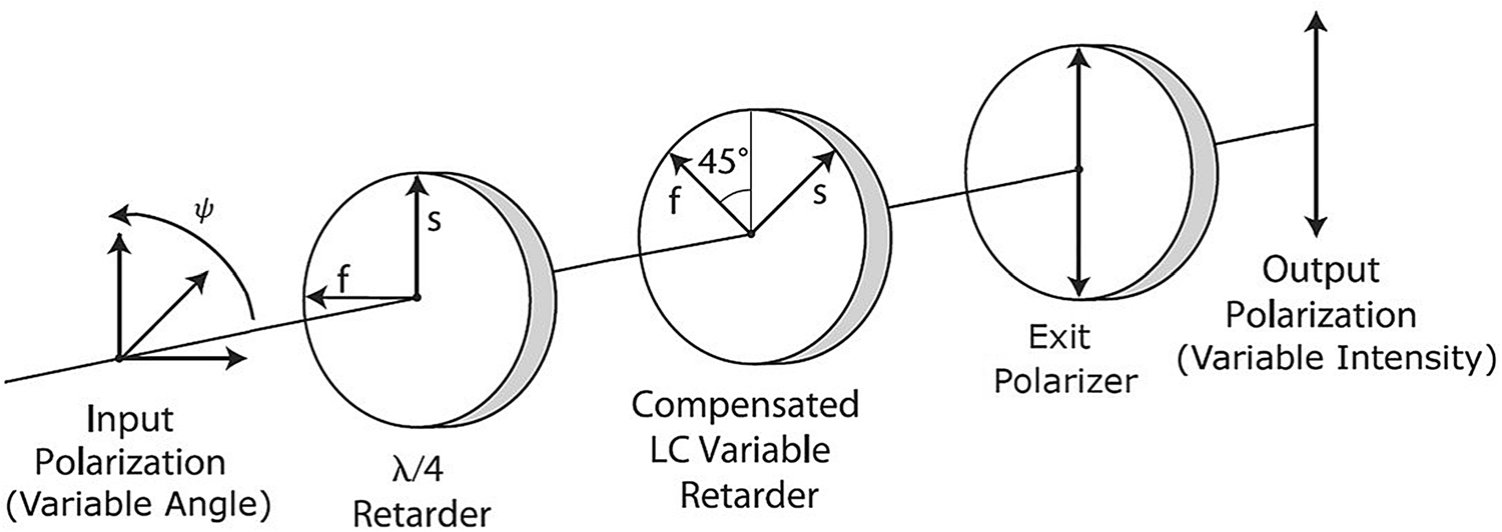
Liquid crystal polarization analyzer scheme.

The scheme of the LCPA is shown in Fig. 2. It consists of a quarter-wave plate, the LCVR and a static polarization filter. Calculations, given later in the “Methods” section, show that the LCPA encodes the input polarization angle $\psi$ into the output light intensity *I* as:

1 $$\begin{aligned} I\left( \delta ,\psi \right) =\cos ^{2}\left( \frac{\delta }{2}-\psi \right) , \end{aligned}$$

where $\delta$ is the retardancy, introduced by the LCVR and $\psi$ is the polarization angle of the incident light. As it can be seen from the formula (Eq. 1) the LCPA selects polarization angles for which the difference $\delta /2-\psi$ is close to $n\pi$. Therefore, varying the retardancy it is possible to probe the polarization distribution of the incident light.

**Figure 3:**
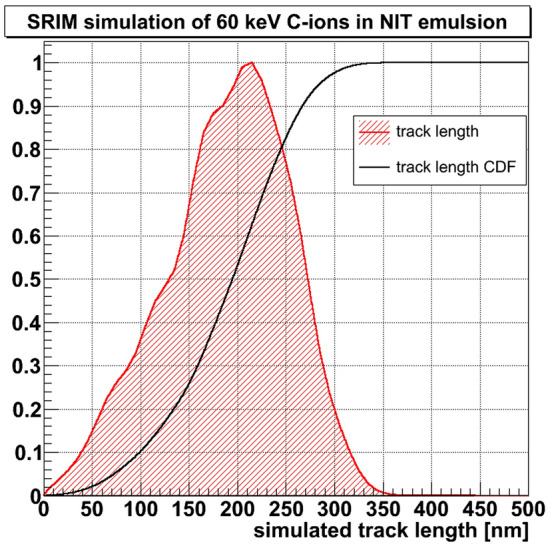
(Solid red curve shaded diagonally) Track length distribution of 60 keV carbon ions in emulsion calculated with SRIM and (black solid line) its cumulative distribution function (CDF).

### Analysis method

As it was mentioned before, silver grains in the NIT emulsion are not spherical and have the form of randomly oriented filaments. Since the illumination system uses monochromatic light, a grain becomes detectable when its reflectance is enhanced by the LSPR phenomenon, i.e. when the plasmon resonance peak wavelength matches that of the illuminating light, which happens only at certain polarization angles. In case of several grains being closer than the optical resolution, due to their filament-like shape and random orientation, the contribution of each grain to the brightness of the detected unresolved image is different for different polarization angles. This effect leads to detectable changes in the shape and position of the observed image of unresolved close grains, making their isolation and measurement possible.

The analysis method is based on probing the intensity of different polarization components of the light scattered by silver grains. Due to polarization anisotropy of the LSPR phenomenon, the scattered light intensity distribution changes with varying the polarization angle, providing access to the nano-scale details hidden beyond the diffraction limit.

In order to test the performance of the designed microscope and the analysis method we used a sample exposed horizontally to carbon ions with energy 60 keV. Carbon ions were implanted in the emulsion films under vacuum in such a way to have reference tracks in the emulsion with the given energy. According to the SRIM simulation, the average path length in the NIT emulsion is equal to 210 nm, with a tail ranging up to 370 nm, as shown in Fig. 3.

**Figure 4:**
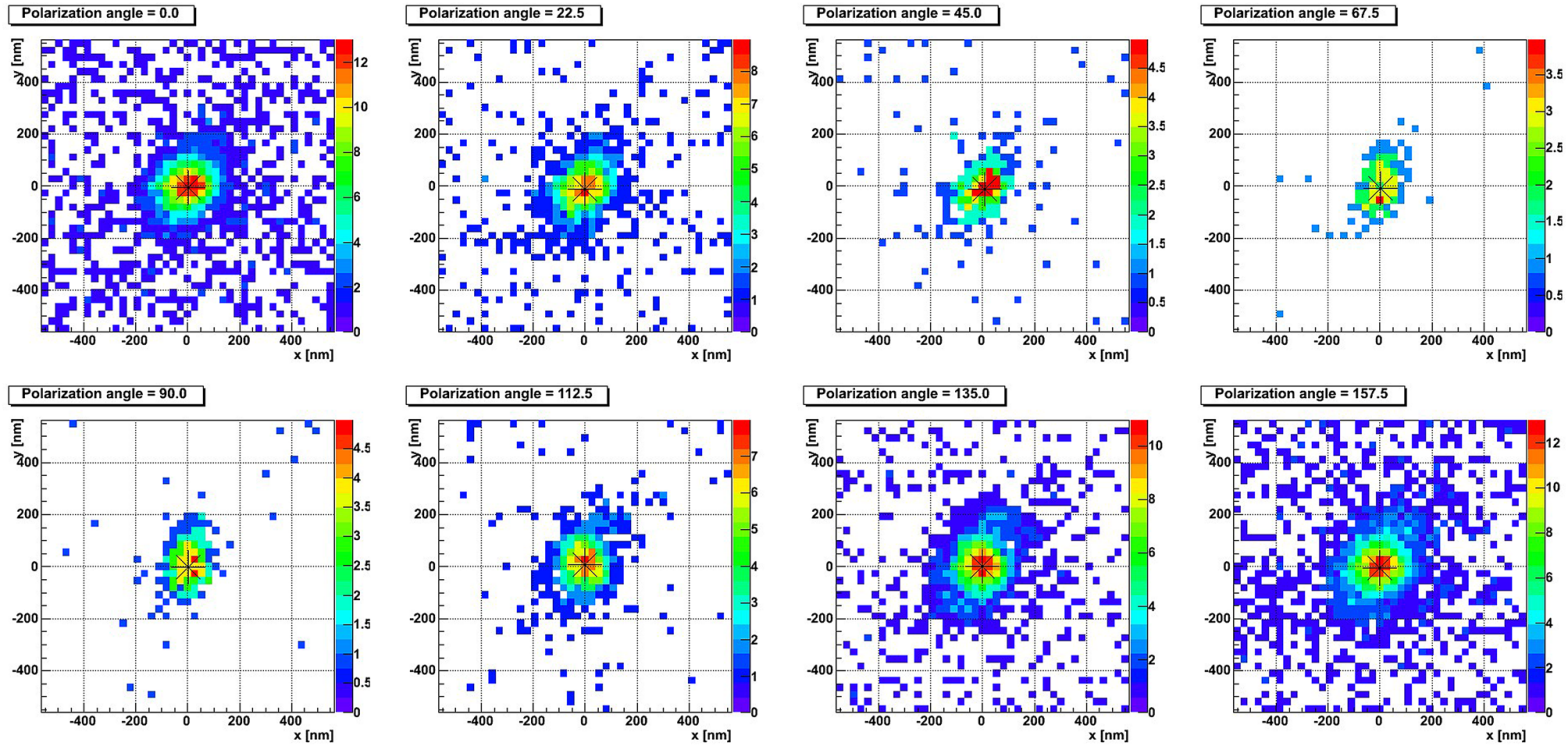
Example of a short barycenter shift event. The color encodes pixel brightness. Barycenter positions are marked with asterisks.

**Figure 5:**
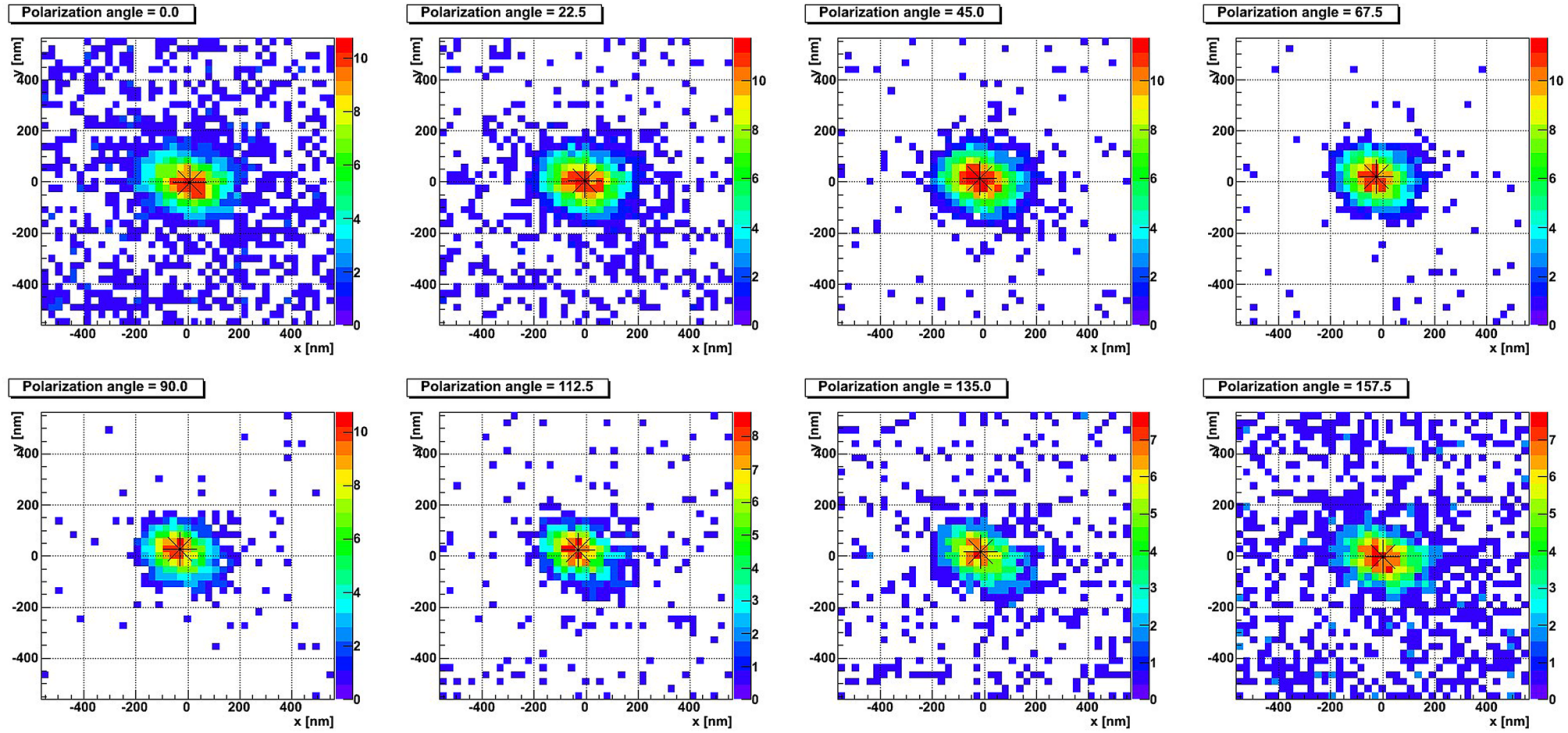
Example of a long barycenter shift event. The color encodes pixel brightness. Barycenter positions are marked with asterisks.

**Figure 6:**
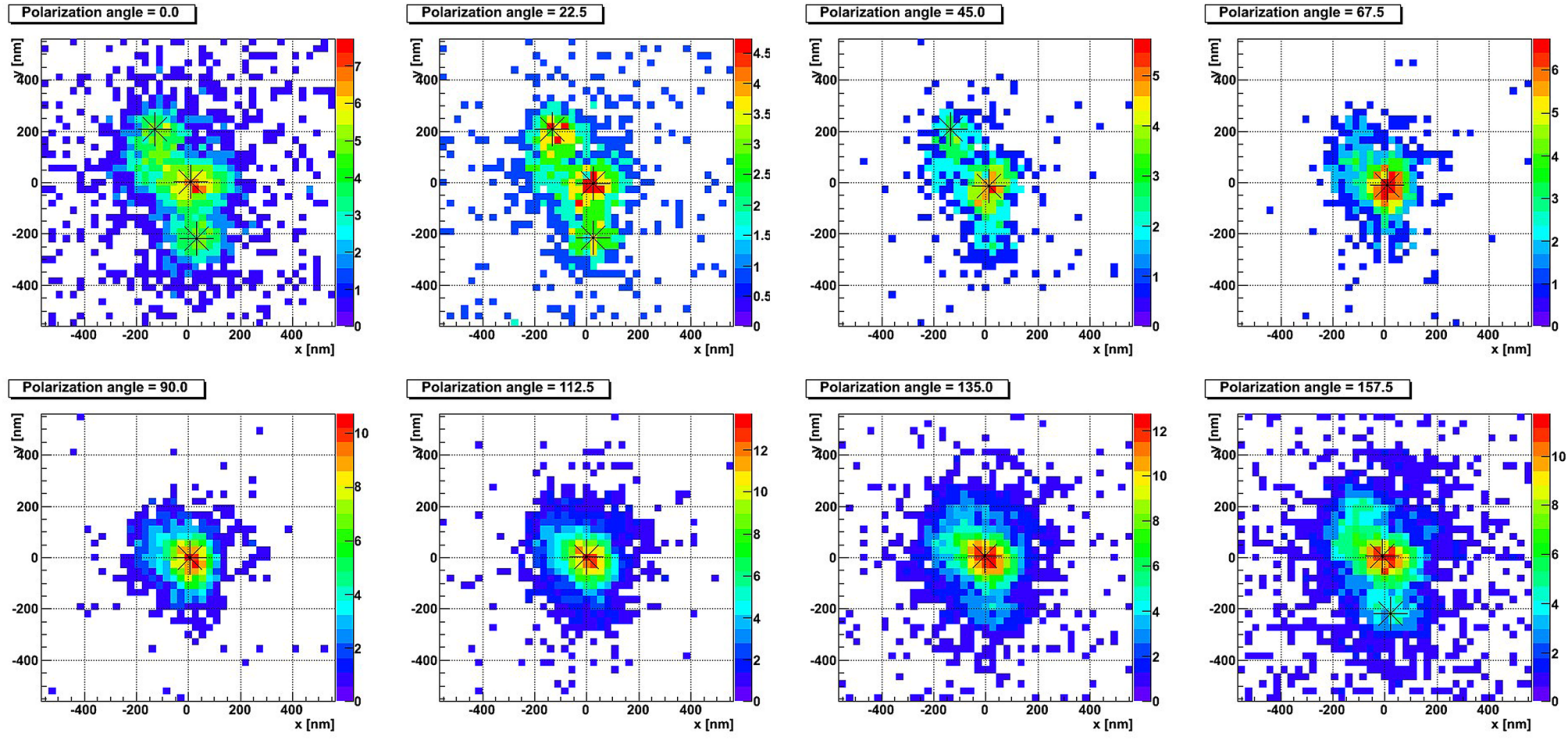
Example of a resolved event. The color encodes pixel brightness. Barycenter positions are marked with asterisks.

Figures 4, 5 and 6 show typical event types observed in NIT emulsions. The eight plots of each figure represent best focused images (also called clusters) taken at different polarization angles of a single grain (Fig. 4), of two unresolved grains (Fig. 5) and of four unresolved close grains (Fig. 6). The eight polarization angles are equally separated by $22.5^{\circ }$. The color scale represents the light intensity of the given pixel for the given polarization angle. The center of black asterisk represents the measured barycenter position.

The spherical shape of the cluster in Fig. 4 changes only marginally with the polarization and its barycenter stays static. On the contrary, the barycenter of the cluster in Fig. 5 noticeably displaces with the polarization angle, as well as its shape changes from spherical to elliptical and then back to spherical. The event in Fig. 6 is resolved: at several polarization angles it looks like two or three distinct clusters, while in other images it is visible as a single elongated cluster.

**Figure 7:**
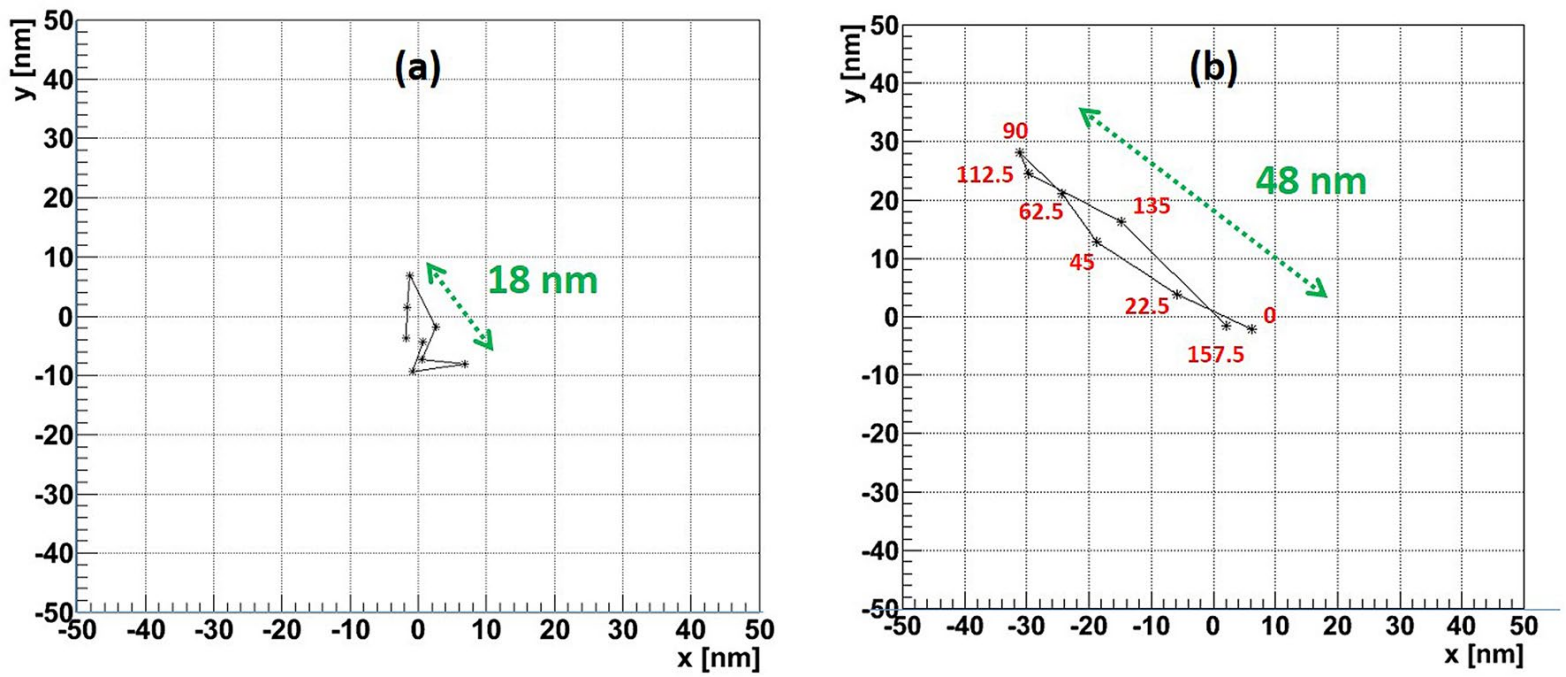
Barycenter displacement profiles for **(a)** the short barycenter shift event from Fig. 4 and **(b)** the long barycenter shift event from Fig. 5.

Figures 7a,b show the barycenter position (black asterisks) of a cluster for each angle of polarization (red numbers nearby) for events shown in Figs. 4 and 5, respectively. We define a *barycenter shift* as a line connecting the most distant points in these plots. Therefore, the barycenter shift length is defined as the length of this line, while the direction is the line’s angle with the X axis. The event from Fig. 7a has the barycenter shift equal to 18 nm, while for the one shown in Fig. 7b it is equal to 48 nm and the direction angle is $37.4^{\circ }$. For resolved events, containing at some polarizations resolved cluster pairs (like in Fig. 6), the direction and the length are taken equal to those of the line connecting the two most distant clusters in resolved images.

It is important to note that the measured value of barycenter shift depends on mutual orientation of grain filaments composing the track: when they are perfectly parallel their brightness changes synchronously and the barycenter shift is minimal, almost close to zero. On the contrary, when they are orthogonal, the barycenter shift is maximal, close to the real length of the horizontal projection of the track. In general case, due to random orientation of filaments, we expect that the barycenter shift is always smaller than the track’s projection at the horizontal plane. Hence, the barycenter shift can be interpreted as a lower limit on the track length.

Direction and length of tracks are of great importance for the directional WIMP search since they are related to the direction, mass and energy or the original WIMP that caused the nuclear recoil, which was recorded as a track. Barycenter shift direction can be interpreted as the direction of the track, but barycenter shift length cannot be considered as track length. Nevertheless, there is a strong correlation between them: tracks cannot produce barycenter shifts longer than their own lengths. Therefore, barycenter shift length can be used to separate events with reliable directional information (long tracks) from those with absent or unreliable directional information (single grains, short tracks). Naturally, a part of long tracks that produces short barycenter shifts will be excluded, thus, lowering the overall sensitivity of the emulsion detector, but the remaining part will contain mainly long tracks suitable for WIMP direction measurement.

**Figure 8:**
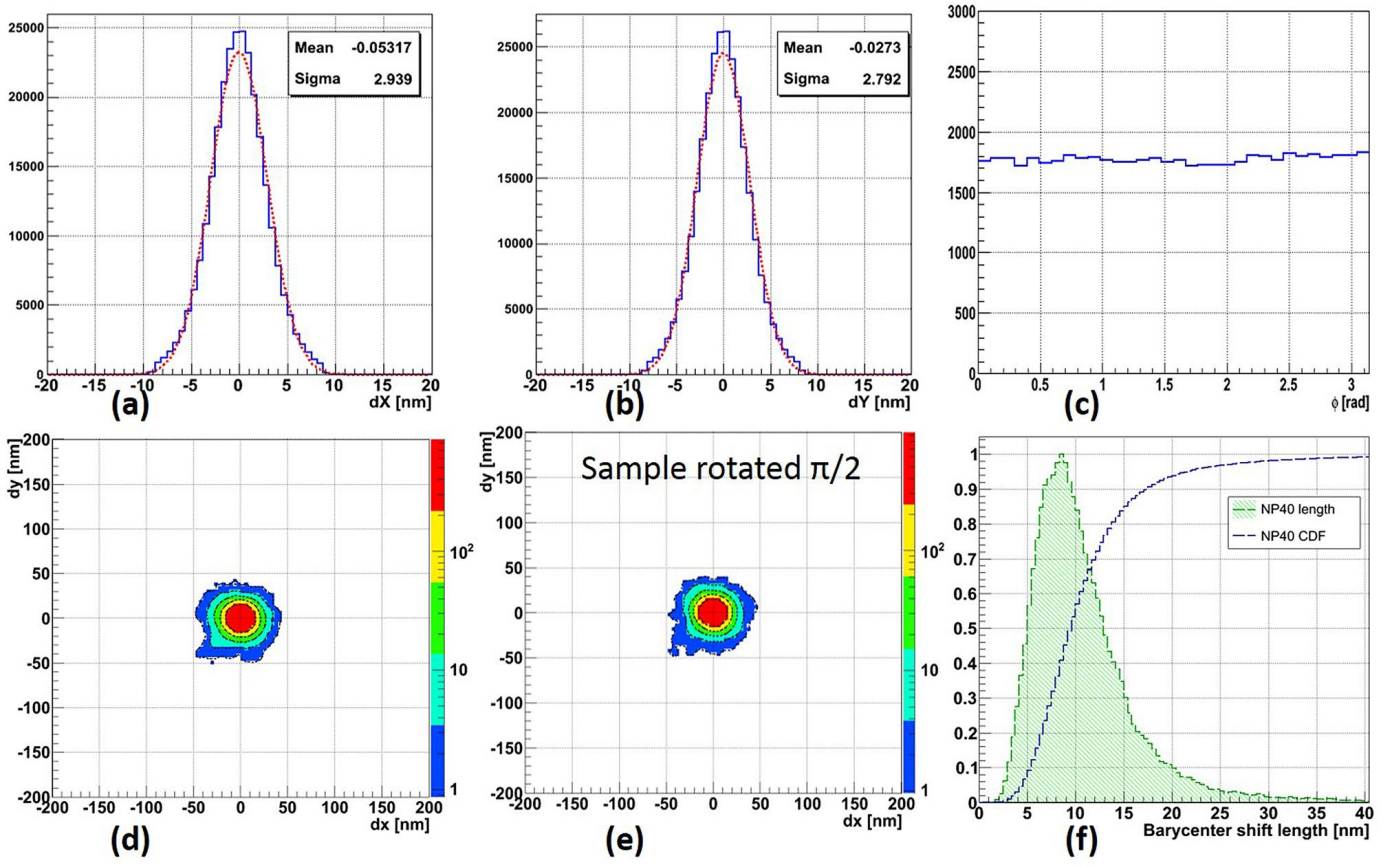
Measured spatial accuracy for **(a)** X and **(b)** Y axes. **(c)** Barycenter shift direction distribution. **(d)** Barycenter shift pattern. **(e)** Barycenter shift pattern for the same sample rotated at $\pi /2$. **(f)** Barycenter shift length distribution (green dashed line shaded diagonally) and its CDF (blue dashed line).

### Spatial accuracy

Though the optical resolution is limited by diffraction, and the size of a silver NP cannot be measured directly, its position can be calculated with rather high precision. However, the spatial accuracy is limited by the presence of thermal noise on the camera sensor and by vibrations induced from outside. Indeed, two consecutive position measurements of the same NP do not yield exactly the same result. Nevertheless, the spatial accuracy can be much higher than the optical resolution. As shown in Fig. 8a,b, the measured spatial accuracy of the reported microscope is slightly better than 3 nm for both axes. The measurement method is described later, in the “Methods” section.

### Microscope calibration

The microscope was calibrated in a way to have an isotropic response to 40 nm spherical silver NPs. The isotropy of angular measurements is confirmed by the flatness of the barycenter shift direction distribution shown in Fig. 8c. The barycenter shift pattern, shown in Fig. 8d, is quite isotropic, as well, except for the outermost region, where the statistics is rather low and measurements are subject to fluctuations. As expected, for spherical NPs the rotation of a sample does not change the barycenter shift pattern, shown in Fig. 8e. From the barycenter shift length distribution, shown in Fig. 8f, follows that 95% of measured NP events are shorter than 21 nm. In our further analysis we use this value as a threshold to distinguish short barycenter shift events, caused by single NPs, from those long, caused by multiple silver grains composing a charged particle track in emulsion.

### Experimental test results

**Figure 9:**
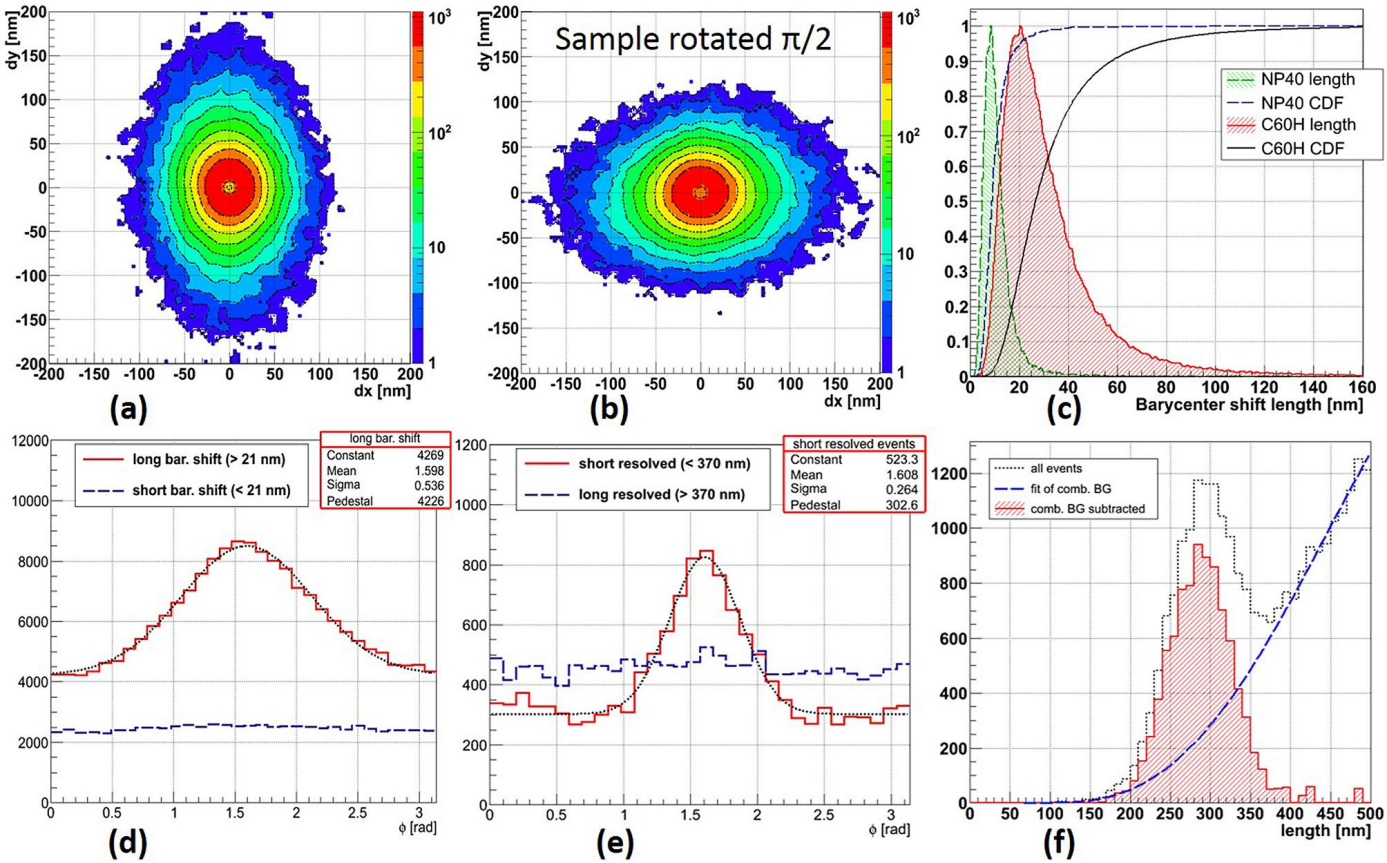
**(a)** Barycenter shift pattern for the sample exposed to 60 keV carbon ions. **(b)** Barycenter shift pattern for the same sample rotated at $\pi /2$. **(c)** Barycenter shift length distributions for (green dashed line shaded diagonally) 40 nm NPs and (red solid line shaded diagonally) and their CDFs (blue dashed line) and (black solid line), correspondingly. **(d)** Barycenter shift angular distribution for the (red solid line) long and (blue dashed line) short fractions. **(e)** Angular distributions of the (red solid line) short and (blue dashed line) long resolved events fractions. **(f)** Length distribution of resolved events: (black dotted line) all resolved events, (blue dashed line) estimated combinatorial background due to too close events and (red solid line shaded diagonally) events after subtraction of the combinatorial background line.

We have analyzed the $3.2\,\hbox {mm}^2$ of the sample exposed to 60 keV carbon ions and applied the analysis method described above. The form of the barycenter shift pattern, shown in Fig. 9a, has become elliptical, with the ellipticity increasing with the barycenter shift length. The direction of the major axis coincides with the beam direction and the rotation of the sample makes the pattern rotate at the same angle as shown in Fig. 9b. The barycenter shift length, which distribution is shown in Fig. 9c, is much larger than that of silver NPs. The short barycenter shift fraction of events does not bear any directional information (see Fig. 9d), while the long one has a distinct peak aligned with the known beam direction. The pedestal of the long barycenter shift events is due to a strong scattering of carbon ions at low energies. Indeed, simulations show that the number of back-scattered events is not negligible.

According to the simulation (Fig. 3), the 60 keV carbon ion beam cannot produce tracks much longer than 360–370 nm. Therefore, similarly to the barycenter shift events, we subdivide resolved events into two fractions: short (shorter than 370 nm) and long (longer than 370 nm). The direction of short resolved events, shown in Fig. 9e, although calculated in a different way, coincides well with the direction of long barycenter shift events, and both directions are in good agreement with the known carbon ion beam direction. The fraction of long resolved events is due to the high density $(2 \times 10^7$ ions/cm$^2$) of the exposed sample: two different implanted ions closer than about 500 nm can be classified as resolved events. Therefore, this fraction has an isotropic angular distribution. Subtraction of the estimated number of chance coincidence events from the resolved event length distribution, as shown in Fig. 9f, yields a distribution with the majority of events shorter than 370 nm, in agreement with the endpoint predicted by the simulation (Fig. 3).

### LSPR-based super-resolution imaging

**Figure 10:**
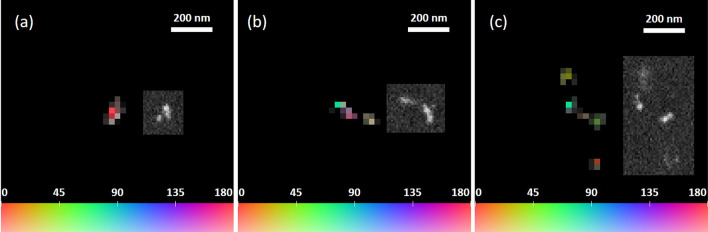
**(a–c)** Reconstructed super-resolution images of events shown in Figs. 4, 5 and 6, respectively. Insets are SEM images of corresponding events. Pixel size in reconstructed images and in SEM images is equal to 27.5 nm and 9.2 nm, respectively. The color in super-resolution images encodes the polarization angle at which the maximum pixel brightness is reached with the scale shown in the bottom of each image. The color saturation level encodes the pixel’s brightness change amplitude caused by the variation of the polarization angle.

The analysis method was verified by scanning the sample with both the reported microscope and a scanning electron microscope (SEM). A super-resolution image is modelled as a set of brightness-modulated pixels, with each pixel being described with three parameters: average brightness, brightness change amplitude and brightness phase (the polarization angle at which the maximum pixel brightness is reached). With this image model a joint deconvolution of event images shown in Figs. 4, 5 and 6 was performed, resulting in images shown in Fig. 10. Corresponding SEM images of events are shown in insets.

The content of the reconstructed images closely corresponds to that of SEM images. The short barycenter shift event in Fig. 10a represents a compact structure containing a single filament (or, feasibly, two extremely close ones). The SEM image of the long barycenter shift event (inset of Fig. 10b) shows two distant filaments with a gap of about 50 nm between them. The reconstructed super-resolution image of the same event, shown in Fig. 10b, contains three different objects. There can be several reasons for this discrepancy. First, due to that only surface events can be studied with the SEM, the silver grain missing in the SEM image could be occasionally removed during the sample preparation for SEM scanning. Second, the electron beam of the SEM is destructive for organic gelatin and can burn bonds fixing silver filaments in place, thus, causing their migration or, even, a complete removal, which results in altering the underlying grain configuration. Due to the necessity of the specific sample preparation for SEM scanning it becomes impossible to repeat the acquisition at the optical microscope. The reconstruction of the resolved event, shown in Fig. 10c reveals four distinct objects, two of which are clearly visible at the SEM image, the other two are most probably located below the surface, out of SEM’s reach, making only their shadows visible in the SEM image. The resolution of reconstructed images is limited by the hardware pixel size and can be estimated by using the Nyquist-Shannon-Kotelnikov sampling theorem as approximately 80 nm.

## Discussion

We demonstrated that the designed microscope and the developed analysis method are capable of isolating tracks produced by low-energy carbon ions in a completely automated mode. According to the simulations, the major part of tracks have the path length shorter than the diffraction-limited resolution of best conventional optical microscopes. With the chosen threshold of 21 nm, events with the barycenter shifts longer than that, represent tracks produced by carbon ions from the beam. Measured barycenter shift direction corresponds to that of the track. The barycenter shift length depends on mutual orientation of grains composing the track and, therefore, its length is not equal to that of the track, unless the grains are perpendicular to each other. The correlation between the barycenter shift and the actual track length will be investigated with electron microscopes and it will be the subject of a dedicated paper.

The reported analysis method has certain limits of applicability. It is optimized for samples in which randomly oriented non-spherical metallic NPs are dispersed in a dielectric media transparent at the illuminating light wavelengths. No barycenter shift will be detected if the two NPs are spherical and also if they are non-spherical but happen to be oriented along the same direction.

Further development of the analysis method can help extracting more information about the nano-scale structure of events. For example, from Eq. (1), since the retardancy $\delta$ is known, it is possible measure the polarization angle $\psi$ that should coincide with the direction of the major axis of a silver grain.

We showed that reported microscope can produce super-resolution images by performing a joint deconvolution of images taken at different polarization angles. The achieved resolution was estimated to be about 80 nm and is limited by the hardware pixel size, thus, leaving room for improvement. A new setup with smaller camera sensor pixels and higher optical magnification is required to reveal the full potential of the LSPR-based super-resolution imaging.

Unlike previous manual implementations^4,5,17^, we managed to minimize the effect of vibrations in the designed super-resolution optical microscope. Therefore, it can be used to analyze isolated events when there is no possibility to apply pattern matching algorithms for vibration correction. Better isolation from vibrations coupled with reducing the camera sensor noise can potentially improve the spatial accuracy and lower the detection threshold. The readout speed of the prototype microscope can be further increased by substituting the LCVR with a Pockels cell that has a faster response to the external electric field. We also evaluate the possibility of splitting the field of view into several identical images and analyze each of them with dedicated polarization filters rotated at different polarization angles. The latter will not only permit to analyze all polarization angles simultaneously, but also will allow to use the white light illumination to extend the analysis to multiple wavelengths. The simultaneous acquisition of polarization images will make the microscope insensitive to vibrations and will enable application of novel scanning techniques^22,23^, dramatically boosting the readout speed.

The combination of the reported method and the iSCAT^24^ technique, with using a coherent optical source, can enable the axial coordinate measurement, thus, providing a full 3D picture of nanostructures under study. Moreover, such a combination can reduce the minimal detectable NP size and will enable the detection of even shorter tracks, resulting in a significant boost of the sensitivity of the NEWSdm nano-emulsion detector.

The creation of a fast and completely automated microscope capable of reconstructing low-energy sub-micron ion tracks in nano-grained emulsions paves the way for construction of a solid-state detector exploiting the directionality as an additional parameter enabling the conclusive identification of WIMP events, even in the presence of background. Moreover, this approach can overcome the neutrino floor, thus, enabling the exploration of the WIMP parameter space unreachable by contemporary dark matter search experiments.

The reported microscope and analysis method can also be used for applications outside the directional dark matter search for fast automated analysis of materials whose optical properties are anisotropic with respect to the polarization direction. For example, it can be used to reconstruct the spatial orientation of NPs that is of great importance for understanding of certain vital biological mechanisms, such as the heterogeneous local structure deformations of the cell membrane, or the interaction force between proteins and cell membranes, etc. It can be used to verify various hypotheses of the antimicrobial effect of silver NPs that requires the study of the mechanism of the cell membrane penetration, research of the interaction with enzymes and the DNA inside the cell and the understanding of the modulation of cellular signal transduction caused by the presence of silver NPs^18^. A possibility would be its application in microfluidics to visualize the orientation of non-spherical metallic NPs inside capillars and near membrane walls, where, due to the arising of hydrodynamic interactions between the particle and the walls, the diffusive behavior of a NP in the presence of confining walls is dramatically different from that occurring in the bulk^19^. Another interesting application could be for the classification and design of magneto-optical materials^20^, where due to the Kerr effect, the polarization of the reflected light depends on the local magnetic state, making the visualization of magnetic domain structures possible with the reported microscope. Moreover, after minor modifications like the adjustment of the illumination light wavelength and the use of a dichroic mirror instead of the beamsplitter, it will become possible to exploit the designed microscope to study the field-dipole interactions of fluorescent dye molecules^21^.

## Methods

### Microscope setup

The prototype microscope, shown in the left panel of Fig. 1, uses a high magnification objective lens with high numerical aperture (Nikon CFI Plan Apo Lambda $100\times /1.45$ Oil). An additional magnification lens in front of the camera makes the total magnification equal to $260\times$. The critical-type illumination system is customly designed and houses a bright blue $(460 \pm 25\, \hbox {nm})$ LED light source (Luminus CBT-120). As shown in the scheme in the right panel of Fig. 1, the microscope is configured to operate in the reflection mode, thus, providing better signal-to-noise ratio. It is equipped with a fast 4 megapixel monochromatic camera (Allied Vision Technologies Bonito CL-400B) working at 100 fps. A liquid crystal polarization rotator device (Meadowlark Optics LPR-200) coupled with a static polarization filter allows analyzing the polarization of the scattered light. The sample can be moved in the horizontal plane by the means of a motorized stage (Micos MS-8), while the vertical movement is achieved by displacing the objective lens, along with the optical system as a whole, with a linear stage (Micos UPM-160). Additionally, the microscope is equipped with pneumatic vibration dumpers (Fabreeka PLM 1).

Microscope components are controlled from a workstation (Dell T7500) equipped with a framegrabber (Matrox Radient eCL), a motion control board (National Instruments PCI-7344) and a GPU board (GeForce GTX 780) for accelerated image processing. The LASSO (Large Angle Scanning System for OPERA) software framework^25,26^ provides modules for real-time microscope automatization, image acquisition and data analysis.

### LCPA formula derivation

The polarization state of incoherent light is described by a Stokes vector. The effect of an optical system on the polarization of light can be determined by constructing the Stokes vector for the input light and convolving it with Mueller matrices representing the optical system. As a result, one gets the Stokes vector of the light leaving the system. The LCPA system can be described mathematically in terms of Mueller matrices as:

$$\begin{aligned} M_{LCPA}(\delta )=\frac{1}{2} \left[ {\begin{array}{cccc} 1 &{} 1 &{} 0 &{} 0 \\ 1 &{} 1 &{} 0 &{} 0 \\ 0 &{} 0 &{} 0 &{} 0 \\ 0 &{} 0 &{} 0 &{} 0 \end{array}}\right] \left[ {\begin{array}{cccc} 1 &{} 0 &{} 0 &{} 0 \\ 0 &{} \cos {\delta } &{} 0 &{} \sin {\delta } \\ 0 &{} 0 &{} 1 &{} 0 \\ 0 &{} -\sin {\delta } &{} 0 &{} \cos {\delta } \end{array}}\right] \left[ {\begin{array}{cccc} 1 &{} 0 &{} 0 &{} 0 \\ 0 &{} 1 &{} 0 &{} 0 \\ 0 &{} 0 &{} 0 &{} -1 \\ 0 &{} 0 &{} 1 &{} 0 \end{array}}\right] =\frac{1}{2} \left[ {\begin{array}{cccc} 1 &{} \cos {\delta } &{} \sin {\delta } &{} 0 \\ 1 &{} \cos {\delta } &{} \sin {\delta } &{} 0 \\ 0 &{} 0 &{} 0 &{} 0 \\ 0 &{} 0 &{} 0 &{} 0 \end{array}}\right] , \end{aligned}$$

where $\delta$ is the retardancy introduced by the LCVR. The three matrices in the middle part of the equation represent Mueller matrices for the exit polarizer plate, the LCVR and the quarter-wave plate, respectively, with their mutual orientation being taken into account. The polarized light scattered from a NP can be expressed by the following Stokes vector:

$$\begin{aligned} S_{in}(\psi )=\left( {\begin{array}{c} 1 \\ \cos {2\psi } \\ \sin {2\psi } \\ 0 \end{array}}\right) , \end{aligned}$$

where $\psi$ is the polarization angle. After passing through the LCPA the light state transforms as:

$$\begin{aligned} S_{out}(\delta ,\psi )=M_{LCPA}(\delta )S_{in}(\psi )=\frac{1}{2} \left[ {\begin{array}{cccc} 1 &{} \cos {\delta } &{} \sin {\delta } &{} 0 \\ 1 &{} \cos {\delta } &{} \sin {\delta } &{} 0 \\ 0 &{} 0 &{} 0 &{} 0 \\ 0 &{} 0 &{} 0 &{} 0 \end{array}}\right] \left( {\begin{array}{c} 1 \\ \cos {2\psi } \\ \sin {2\psi } \\ 0 \end{array}}\right) = \left( {\begin{array}{c} \cos ^{2}(\frac{\delta }{2}-\psi ) \\ \cos ^{2}(\frac{\delta }{2}-\psi ) \\ 0 \\ 0 \end{array}}\right) . \end{aligned}$$

The first component of the resulting Stokes vector is the intensity registered by the camera sensor and is, therefore, the Eq. (1).

### Data acquisition parameters

The data acquisition is performed by scanning the entire detector volume with samplings of 60, 43 and $0.25 \upmu \,\hbox {m}$ along X,Y and Z axes respectively. The X and Y samplings are chosen to be almost equal to the corresponding field of view dimension, leaving about $5 \upmu \,\hbox {m}$ of overlap to enable the reconstruction of edge events. The vertical sampling, equal to 250 nm, is chosen to be approximately a half of the objective’s depth of field $(\sim 500 \,\hbox {nm})$. Therefore, any grain is seen focused at least in one frame. The polarization angle sampling is chosen as $22.5^\circ$ to cover the full angular range with 8 images.

The readout is performed in the following manner:

1. In static position, take 8 images varying the polarization angle;
2. If still inside the sample make a step along Z axis, otherwise move along Y or X;
3. Repeat (1) and (2) until the whole sample volume is scanned.

The readout speed achieved with these parameters for the reported samples is about 10 s/view.

### Image processing

Before the processing, an image of static background is prepared by grabbing a series of images in the absence of a sample. Then, a single image is generated by averaging values of corresponding pixels of the images grabbed during the previous step. The averaging leaves only static features present in images, like dead pixels and images of dust particles attached to the camera sensor as well as the non-uniformity of the illumination brightness level across the image.

The first step of image processing involves the subtraction of the pre-generated static background image from every image grabbed, making the illumination brightness distribution flat, removing static image features and leaving only the features belonging to the signal.

In the next step, a flattened image is processed with a $9\times 9$ convolution filter that enhances image features with dimensions close to the microscope’s optical resolution. The filtration procedure is GPU accelerated. The details can be found in ref.^27^ and references therein.

### Cluster reconstruction

A cluster is a tie set of adjacent pixels corresponding to grain’s image projected on the camera sensor. To isolate individual clusters a threshold function is applied to pixels of a filtered image. Tie sets of adjacent pixels with brightness values higher than the predefined one form eventual clusters.

### 2D Gaussian fit

After a cluster is isolated, the corresponding pixels are extracted from the original flattened image along with pixels from the surrounding region of approximately $80\times 80$ pixels. Then the cluster shape is evaluated by performing the bi-dimensional Gaussian fit with the function *f*(*x*, *y*) to measure the barycenter coordinates $(x_0, y_0)$, lengths along major and minor axes $(\sigma _x, \sigma _y)$ and the angle $\theta$ between the major axis and the X axis:

$$\begin{aligned} f(x,y)=A\exp (-(a(x-x_0)^2+2b(x-x_0)(y-y_0)+c(y-y_0)^2)), \end{aligned}$$

where $$a=\frac{\cos ^2\theta }{2\sigma _x^2}+\frac{\sin ^2\theta }{2\sigma _y^2}$$, $$b=-\frac{\sin 2\theta }{4\sigma _x^2}+\frac{\sin 2\theta }{4\sigma _y^2}$$ and $$c=\frac{\sin ^2\theta }{2\sigma _x^2}+\frac{\cos ^2\theta }{2\sigma _y^2}$$.

### Grain reconstruction

Since the vertical sampling is finer than the depth of field, a single physical grain produces multiple clusters: not only the in-focus one but also those out-of-focus. In order to isolate all clusters belonging to the same grain, a starting cluster is linked with clusters at the adjacent frames that have an offset in XY less than 300 nm. Then, this procedure is repeated for the just linked clusters until there is no more clusters to link. As a result of this procedure, one gets a tree-like collection of clusters that is associated with a grain or, more general, with an event. Thus, an event can contain a single grain, a close unresolved grain pair (a track) or a close resolved grain pair (a microtrack).

### Best focused cluster selection

The barycenter shift analysis procedure is defined only for in-focus clusters and, therefore, it is necessary to find the best focused cluster (BFC) inside the clusters collection tree representing a single grain or an event. A cluster with the highest amplitude, found by the bi-dimensional Gaussian fit, is selected as the BFC by iterating all clusters in the collection.

### Spatial accuracy measurement method

The data acquisition is performed in the same way and with the same parameters as for real measurements. Eight images corresponding to different polarization angles spaced by $22.5^\circ$ are taken at every step. After that, the image processing, cluster and grain reconstruction are carried out. Then, for each set of eight clusters corresponding to the same grain and belonging to the best focus plane of that grain, one plots the difference of the cluster’s barycenter with its arithmetic mean over all polarizations. Combining these plots for each axis, one gets the spatial accuracy plot for the given axis, as shown in Fig. 3.

### NIT samples description and exposure details

Nuclear emulsions are made of small crystals of silver halide (usually bromide, AgBr) immersed in an organic gelatin^8^. The energy loss of ionizing particles crossing the films induces along its path atomic-scale perturbations that, after a chemical treatment, produce a sequence of visible grains in the emulsion. A new type of nuclear emulsion the “Nano-Imaging Tracker” (NIT)^9^ was developed at Nagoya University (Japan) in 2010 intentionally for the directional dark matter search application. The NIT emulsion has grains of 44 nm diameter, respectively, which is about one order of magnitude smaller than the crystal size of the emulsion used in the OPERA experiment^28^. The overall density is equal to $3.44\,\hbox {g}/\hbox {cm}^3$. The granularity corresponds to the average distance between crystals, which is equal to $71 \pm 11\,\hbox {nm}$^9^. NIT emulsion film was exposed to carbon ion beam of 60 keV at the ion implantation system (NH-20SR-WMH) of the Nagoya University Nano fabrication Platform. The beam during the exposure had angle of $10^\circ$ with the emulsion film surface.

## Data Availability

The datasets generated and analysed during the current study are available from the corresponding author on reasonable request.
